# Low-intensity pulsed ultrasound attenuates replacement root resorption of avulsed teeth stored in dry condition in dogs

**DOI:** 10.1038/s41598-021-92471-x

**Published:** 2021-06-18

**Authors:** Saemi Seong, Dohyun Kim, Dasun Lee, Hyung-Ryong Kim, Yooseok Shin

**Affiliations:** 1 Department of Conservative Dentistry and Oral Science Research Center, Yonsei University College of Dentistry, Seoul, 03722 Republic of Korea; 2 College of Dentistry, Dankook University, Cheonan, 31116 Republic of Korea

**Keywords:** Oral diseases, Dental diseases, Dental equipment, Dental trauma, Resorption, Experimental models of disease, Preclinical research

## Abstract

This study aimed to investigate the effects of low-intensity pulsed ultrasound (LIPUS) on replacement root resorption after replantation of avulsed teeth stored in a dry condition in dogs. A total of 73 premolar roots from four male mongrel dogs were intentionally avulsed with forceps and divided into four groups—HN, HL, DN, and DL—according to storage conditions and whether or not they received LIPUS treatment. Thirty-eight roots were kept in Hanks’ Balanced Salt Solution for 30 min (HN and HL groups), whereas the remaining 35 roots were left to dry in the air for an hour (DN and DL groups) prior to replantation. Following replantation, the roots in the HL and DL groups (21 and 18 roots, respectively) received a 20-min daily LIPUS treatment for 2 weeks. The animals were euthanized 4 weeks after the operation. Micro-computed tomography images were acquired for each root and the amount of replacement root resorption was measured three-dimensionally. Histological assessments were also carried out. There was significantly less replacement root resorption for the roots in the DL group compared to the DN group (*p* < 0.01). Histological findings in the DN group demonstrated evident replacement root resorption, whereas the DL group revealed less severe resorption compared to the DN group. Within the limitations, these results suggest that LIPUS could attenuate the replacement resorption of avulsed teeth stored in a dry condition, thereby improving their prognosis.

## Introduction

Tooth avulsion affects 0.5–16% of traumatic dental injuries in permanent dentitions and is considered to be the most serious type of them^1^. Both pulp and periodontal tissues suffer from extensive damage during an extra-alveolar period, with healing reactions dependent upon various factors such as storage media, length of the extra-alveolar period, root development, and contamination of the root surface^2–4^. While tooth replantation is the indicated treatment for some of the avulsions, external root resorption is seen as a major complication encountered after replantation. A meta-analysis on the incidence of root resorption after the replantation of avulsed teeth showed that the incidence of surface, inflammatory, and replacement root resorption was 13.3%, 23.2%, and 51.0%, respectively^5^. Surface root resorption can be seen in a normal healing process and considered as a favorable type of periodontal healing. Inflammatory root resorption is associated with necrosis and infection of the pulp. Replacement root resorption appears as a fusion between the tooth and the alveolar bone, and is related to the condition of periodontal ligaments cells from the injured root surface. The periodontal healing after replantation of avulsed teeth have been the subject of numerous experimental studies. A series of histological analyses of avulsed human teeth showed that almost half of the periodontal ligament was lost after replantation^6^. This indicates serious healing complications that may result in replacement root resorption being commonly observed in avulsed teeth after replantation. This has been confirmed by experimental replantation in dogs^7^, monkeys^8,9^, and humans^10^. Many studies were conducted to improve regenerative responses and healing of periodontal tissues to minimize replacement root resorption after replantation of avulsed teeth. Preventive methods to reduce root resorption susceptibility were also introduced^11^. However, a reliable therapeutic protocol has not yet been universally established.

The use of ultrasound for medical applications has been investigated for decades^12^. Low-intensity pulsed ultrasound (LIPUS) is a specific type of ultrasound that delivers impulses at a low-intensity and outputs pulsed waves. The effectiveness of LIPUS treatments is mostly ensured by non-thermal effects, including acoustic cavitation and biological signaling^13^. LIPUS treatment has been mostly used in the field of orthopedics. It has been shown that LIPUS enhances and accelerates fracture-healing through increased bone formation in cases of delayed and/or impaired bone healing, and significantly decreases period of bone healing^14,15^. Its applications for orthopedic treatment have been approved by the US Food and Drug Administration (FDA) in 1994 and 2000^16^. Although the impact of LIPUS in the osteogenic responses of the bone has been well documented, elucidating the clinical effectiveness of LIPUS in orofacial regions warrants further study. Soft tissue healing and significant decrease in areas of root resorption lacunae have been reported to this date^17,18^. It was found that the application of LIPUS for sinus augmentation promotes new bone formation in rabbits^19^. A pilot study demonstrated enhanced alveolar bone healing of extraction sockets^20^, while another showed the prevention of bisphosphonate-related osteonecrosis of the jaw, both of which were carried out in a rat model^21^. Recently, it was found that LIPUS treatment not only enhances the migration of periodontal ligament stem cells in vitro^22^, but also accelerates 2-wall alveolar bone defects in canines, in addition to inhibiting root resorption after luxation and immediate replantation in rats^18,23^.

A few more studies have been carried out in relation to orthodontically-induced root resorption. It has been reported that LIPUS treatment minimized orthodontically-induced inflammatory root resorption caused by torque in rats^24^ as well as in healthy human patients^25^. It has been shown that LIPUS treatment increases the thickness of predentin and cementum in rats^26^ and beagles^27^. It is considered that LIPUS regulates osteoclast differentiation and bone remodeling^28^. However, no study to date has evaluated the effects of LIPUS on root replantation after avulsion solely dependent on periodontal healing, excluding the inflammatory effects due to infected pulp tissues, which would more accurately reflect the effects of LIPUS on replacement root resorption of avulsed teeth. Thus, this study aimed to investigate the effects of LIPUS on replacement root resorption after replantation of avulsed dog teeth stored with different conditions and periods, excluding the adverse effects from necrotic pulp. It was hypothesized that there would be no difference in replacement root resorption between the roots exposed to LIPUS treatment and those receiving no supplementary treatment after replantation.

## Methods

### Animals

Four healthy male mongrel dogs, 10–14 months of age and weighing approximately 25–30 kg, were included in this study. The animals were housed and monitored daily for the duration of the study in the Department of Laboratory Animal Resources, Yonsei Biomedical Research Institute, Seoul, Republic of Korea. They were allowed a week for acclimation prior to the experiment. They were kept in individual cages at 22 °C with relative humidity of 50%, and a 12-h light/dark cycle. Approximately 500 g of solid food (Purina; Nestle SA, Vevey, Switzerland) was provided to each animal every day, and water was available ad libitum during the experimental period. Experiments were carried out in compliance with the ARRIVE guidelines, approved by the Yonsei University Health System Institutional Animal Care and Use Committee, Seoul, Republic of Korea (Approval No. 2019-0068). Informed consent was obtained from the owners, and all experiments were performed in accordance with relevant guidelines and regulations.

### Surgical procedures

Premolar teeth of each animal were included for the experiment. The roots were divided into four groups according to storage conditions before replantation and whether or not they received a LIPUS treatment after replantation. The roots in each group were evenly distributed among the four dogs, such that each group equally included the right and left, maxillary and mandibular premolar roots. Congenital missing, impacted, or root-fractured teeth were excluded from the study. Thirty-eight roots were kept in Hanks’ Balanced Salt Solution (HBSS) for 30 min (HN and HL groups), whereas the remaining 35 roots were left to dry in the air for an hour (DN and DL groups) before the replantation. The roots in the HL and DL groups received a daily LIPUS treatment after the replantation (Table 1).

**Table 1:** Allocation of roots for each group.

	LIPUS treatment	No LIPUS treatment	Total
HBSS storage (30 min)	21 (group HL)	17 (group HN)	38
Dry storage (60 min)	18 (group DL)	17 (group DN)	35
Total	39	34	73

Three experienced dental clinicians performed all surgical procedures under general and local anesthesia under aseptic routines. General anesthesia was induced by alfaxalone (Alfaxan; Jurox Pty Ltd., Rutherford, NSW, Australia), medetomidine hydrochloride (Tomidine; Provet Ltd., Istanbul, Turkey) intravenously and was maintained by 2% isoflurane (Ifran Liq; Hana Pharm Co Ltd., Republic of Korea) in conjunction with pure oxygen by inhalation.

**Figure 1:**
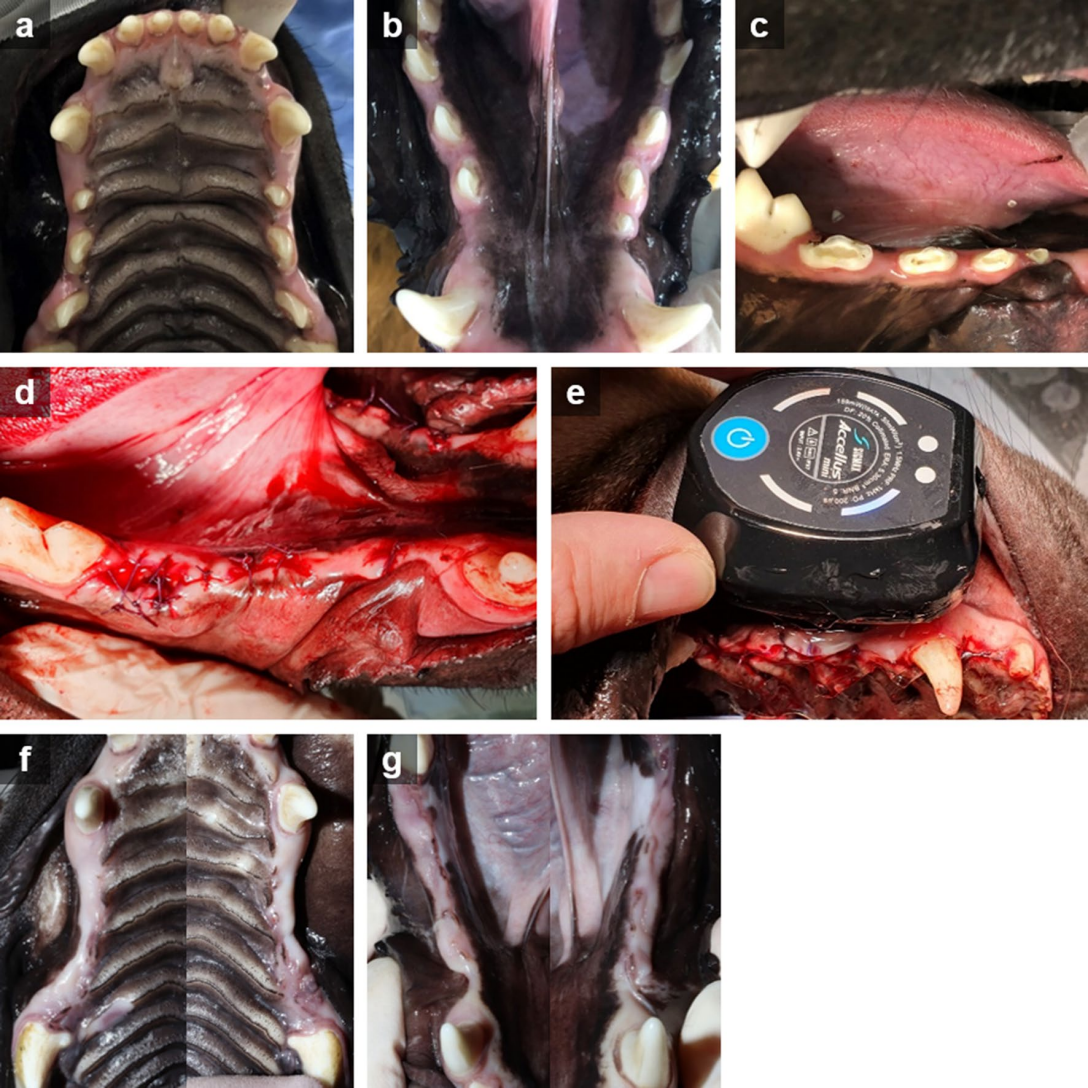
Clinical photographs during experimental periods. (**a**, **b**) Pre-operation. (**c**) After root canal treatment. (**d**) Post-operation. Replanted roots were covered by gingiva and sutured. (**e**) LIPUS treatment (**f**, **g**) 4 weeks after the operation.

Preoperative root canal treatments were performed aseptically on all experimental teeth to minimize the effects of pulpal infection as a stimulus for external root resorption. After occlusal reduction and access opening with sterile diamond bur, coronal flaring was carried out using Gates-Glidden drills (Dentsply Maillefer, Baillagues, Switzerland). The working length was determined using size #20 stainless steel k-type hand instruments (Dentsply Maillefer, Baillagues, Switzerland) and a periapical radiograph. Nickel-titanium rotary instruments (Profile; Dentsply Maillefer, Baillagues, Switzerland) and k-type hand instruments were used for pulp extirpation and root canal preparation to apical delta. The apical enlargements of root canals were accomplished up to size #50–70 with k-type hand instruments. After shaping of the root canal was completed, final irrigation was performed using 5 mL of 2.5% NaOCl solution using a syringe with a 30-gauge needle. Lastly, the root canals were dried with sterile paper points and obturated with Vitapex paste (J Morita, Tokyo, Japan). Occlusal access holes were filled with resin-modified glass ionomer (Fuji II LC; GC, Tokyo, Japan) (Fig. 1a–c).

An intentional avulsion was performed as atraumatically as possible with forceps (Physics Forceps; GoldenDent, Detroit, MI, USA) and gentle bucco-lingual luxation forces. After extraction, two-rooted premolars were hemisectioned into two single roots. The crown portion of the tooth was removed at the cemento-enamel junction with a high-speed diamond bur to avoid occlusal forces during healing periods. Each root was placed in its prepared designated space in a 16-well plate. For the HN and HL groups, each well was filled with 2.5 mL of HBSS, ensuring that the root was completely submerged in the solution for 30 min. For the DN and DL groups, the root was swiftly air dried and then placed in an empty well to dry for an hour. After the given time, the roots were replaced in the socker in their own position and sutured with 4-0 Coated Vicryl (Ethicon Inc, Sommerville, NJ, USA) such that each root was adequately covered with gingiva (Fig. 1d).

**Figure 2:**
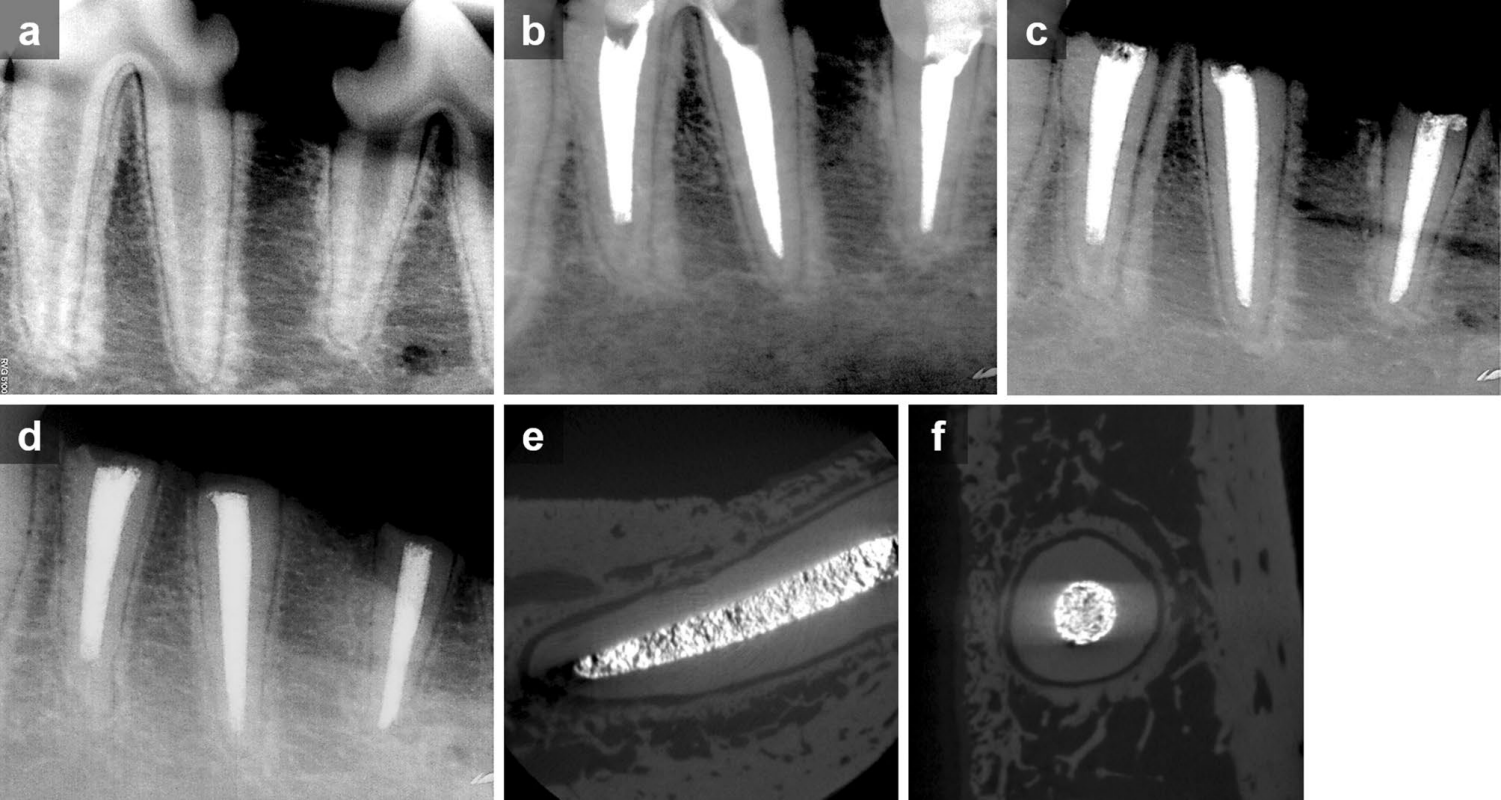
Representative serial radiographs and micro-CT images of roots in the HL group. (**a**) Pre-operation. (**b**) After root canal treatment. (**c**) After replantation. (**d**–**f**) 4 weeks after the operation. Note that the roots were surrounded by intact lamina dura.

**Figure 3:**
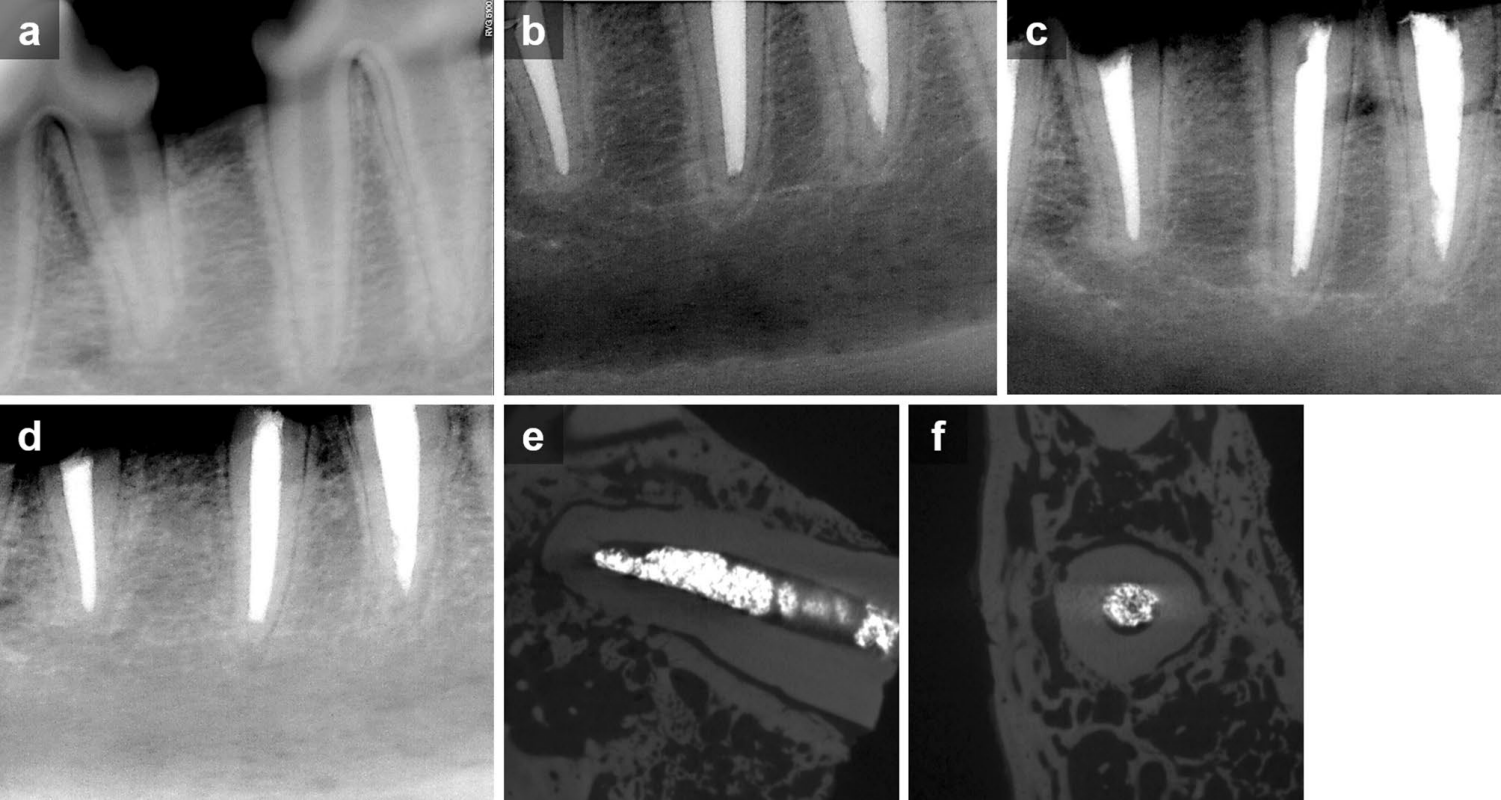
Representative serial radiographs and micro-CT images of roots in the DN group. (**a**) Pre-operation. (**b**) After root canal treatment. (**c**) After replantation. (**d**–**f**) 4 weeks after the operation. Note that the lamina dura became indistinct and the evidence of replacement root resorption was seen in the micro-CT images.

Periapical radiographs were taken before the operation, after the root canal treatment, and after the replantation to confirm each procedure, respectively (Figs. 2a–c and 3a–c). After the surgical procedures, the animals were administered ketorolac thromethamine (Keromin inj; Hana Pharma Co Ltd., Republic of Korea), Cefazolin sodium (30 mg/kg, Chonkundang, Republic of Korea), Meloxicam (0.2 mg/kg, Boehringer Ingelheim, Greece) for a week.

### LIPUS exposure

After replantation, the roots in the HL and DL groups received LIPUS treatment starting from the day of surgical procedure for 2 weeks (Fig. 1e). The LIPUS instrument used in this study (Accellus Mini; Nippon Sigmax Co, Tokyo, Japan) consisted of a circular transducer with a diameter of 26 mm and an effective radiating area of 5.3 cm^2^. The pulsed ultrasound signal had a 1.5 MHz frequency, 30 mW/cm^2^ ultrasound intensity, and 1 kHz pulse repetition rate. For daily ultrasonic exposure, intravenous general anesthesia was delivered to each animal. The transducer was placed in contact with the buccal gingiva, in the region corresponding to the designated quadrant. A single practitioner located the device on the buccal mucosa to cover the targeted area. A coupling gel was kept constantly in place to optimize penetration of the ultrasound energy into the tissues. The ultrasound was used for 20 min daily for 14 consecutive days.

### Three-dimensional (3D) analysis

Four weeks following the operation (Fig. 1f,g), the dogs were euthanized under general anesthesia using an overdose of potassium. After taking periapical radiographs for all roots (Figs. 2d and 3d), both maxillary and mandibular premolar areas were removed en bloc with the surrounding hard tissues and fixed in 10% buffered paraformaldehyde.

**Figure 4:**
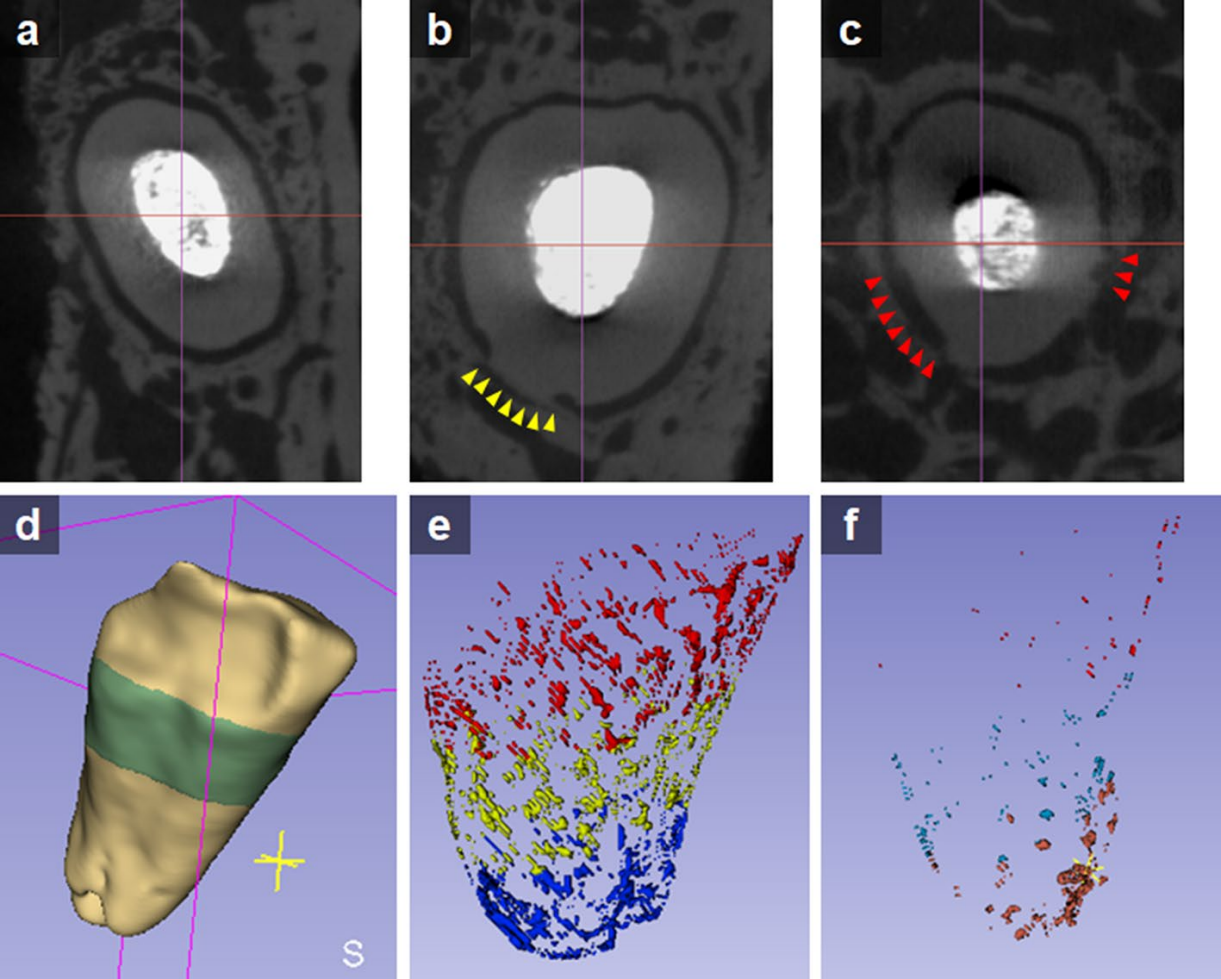
Representative micro-CT images and 3D reconstruction for quantitative measurement of the amount of root resorption. The root embedded in the alveolar bone alone was included. (**a**) Normal root surface without any root resorption. Regular and continuous radiolucent areas between the root surface and the alveolar bone, indicating PDL space, can be seen. (**b**) Replacement root resorption presenting direct connection between the root surface and the alveolar bone without interposition of a radiolucent area (yellow arrowheads). (**c**) Surface root resorption presenting craters or holes in the root surface (red arrowheads) (**d**) Visualization of the root by 3D reconstruction. (**e**) Distribution of replacement root resorption. (**f**) Distribution of surface root resorption.

The blocks were attached in a custom attachment and scanned by a micro-computed tomography (micro-CT) imaging system (Quantum GX; PerkinElmer, Hopkinton, MA, USA) at an isotropic resolution of 40 μm. The images of each sample were quantitatively assessed with regard to both replacement root resorption and surface root resorption. In each coronal cross-section image (Fig. 4a), absence of radiolucent area between the alveolar bone and the root surface was measured for replacement root resorption (Fig. 4b) and distribution of craters or holes in the root surface were measured for surface root resorption (Fig. 4c) using MeshLab^29^ 2020 (https://www.meshlab.net/). Images of each specimen were reconstructed with 3D Slicer^30^ 4.11.0 (https://www.slicer.org/) that provided transaxial, coronal, and sagittal cross sections and visualization, including volume rendering of the samples (Fig. 4d). The areas of replacement and surface root resorption were reconstructed three-dimensionally (Fig. 4e,f), and the percentage of replacement and surface root resorption to whole root surface were calculated respectively.

### Histologic evaluation

The specimen blocks were decalcified in 10% ethylenediaminetetraacetic acid solution (pH 7.4) at 4 °C for 4 weeks embedded in paraffin. Serial, 3 μm-thick sagittal sections of selected roots were cut including the surrounding tissue. For histological analyses, the sections were stained with hematoxylin and eosin (H & E) or tartrate-resistant acid phosphatase staining of osteoclasts (TRAP) and observed under light microscopy.

### Statistical analysis

The percentages of surface and replacement root resorption were compared among the four groups using a one-way analysis of variance followed by a Tukey’s post-hoc test. All statistical analyses were performed under a 95% confidence level using the SPSS 25 (IBM Corp, Somers, NY, USA) software program.

## Results

### Postoperative clinical observations

Postoperative clinical healing was uneventful at all sites. Pus discharge from the gingiva was noticed in some experimental teeth with buccal bone fracture at 2 weeks, which were excluded. No visible adverse reactions, including suppuration, abscess formation or increased tooth mobility, were observed in all roots after 4 weeks.

### Amounts of root resorptions

**Figure 5:**
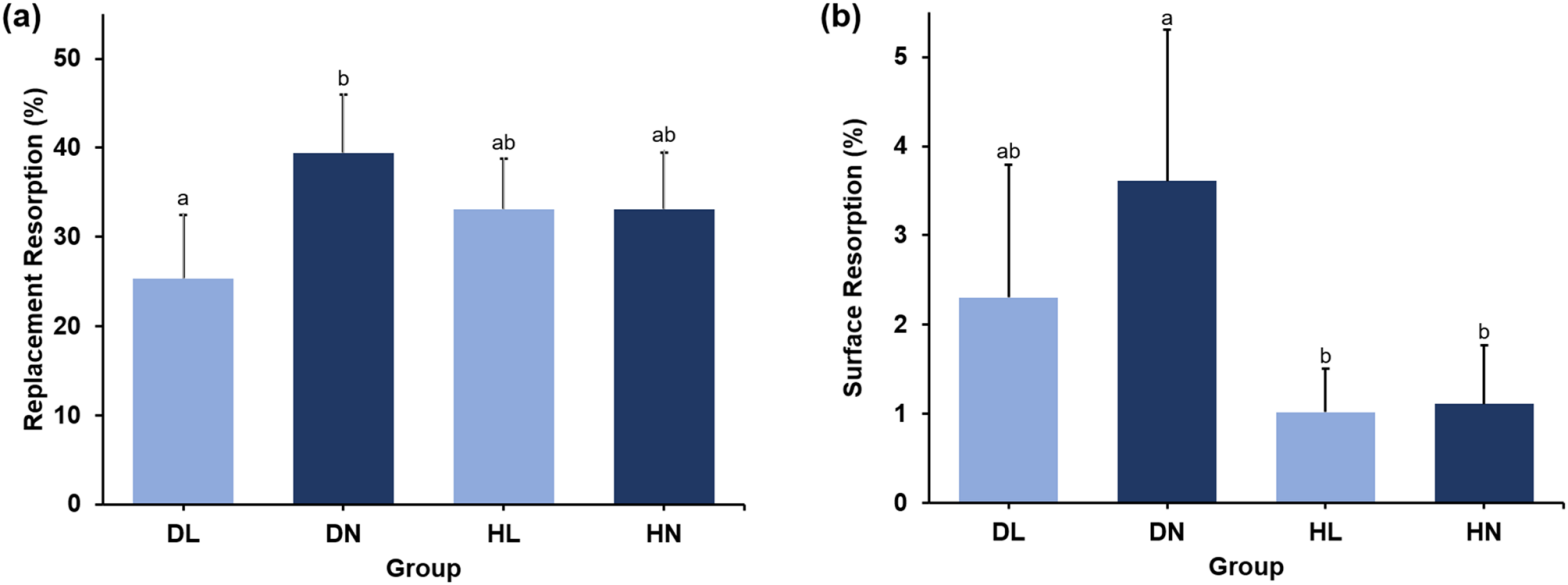
Percentage of root resorption area in each group. (**a**) Replacement resorption. (**b**) Surface resorption. Different letters denote significant different among the groups (p < 0.05).

In dry storage conditions, there was significant less replacement root resorption in the LIPUS-treated group (DL) compared to the non-treated group (DN) (*p* < 0.01). Neither the DN nor the DL group was significantly different from the HL or HN group. However, the roots in the DN group tended to have the largest percent of replacement resorption, while those in the DL group had the least (Fig. 5a). The representative radiographs and micro-CT images of roots in the HL and DN groups are shown in Figs. 2 and 3, respectively.

Both HBSS storage groups (HL and HN) had significantly reduced surface resorption compared to the DN group, regardless of LIPUS exposure (*p* < 0.01 and *p* < 0.05, respectively). There were no significant differences between the HBSS treated groups. Within the dry storage groups (DL and DN), the roots in the DL group showed decreased surface resorption compared to those in the DN group. However, there was no statistically significant difference. In addition, the DL group demonstrated no significant differences in surface root resorption compared to the HL or HN groups (Fig. 5b).

### Descriptive histological results

**Figure 6:**
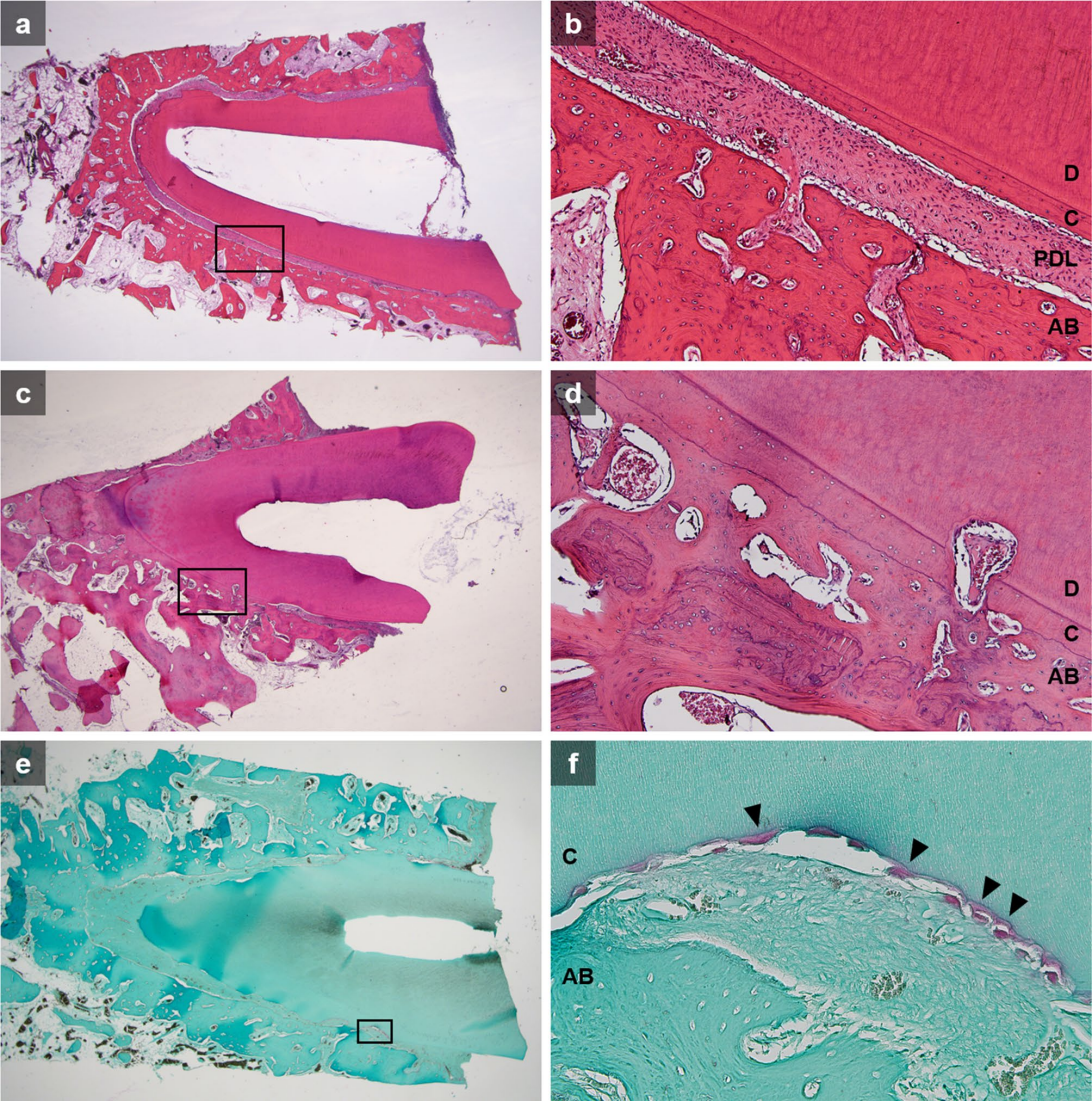
Histologic overview of sections of the replanted roots. (**a**, **b**) A root in the HL group, pointing to minimal root resorption. Note that normal periodontium organized PDL with collagen fibers and cementum layer. (**c**–**f**) A section of root in the DN group, showing replacement root resorption. (**e**, **f**) TRAP staining showed osteoclastic cells (black arrows) confined to the cementum surface. (*D* dentin, *C* cementum, *PDL* periodontal ligament, *AB* alveolar bone).

Cross sections were evaluated for healing according to the criteria of Andreasen^31^. For most roots in the HL and HN groups, the cementum integrity could be observed with periodontal ligament (PDL) space preserved with relatively uniform thickness, where the root was clearly separated from the alveolar bone (Fig. 6a,b). In contrast, histological findings in the DN group demonstrated evident replacement root resorption, where the cementum of the root and alveolar bone was connected, with no clear border in between (Fig. 6c,d). Osteoclastic cells were observed through TRAP staining as well (Fig. 6e,f). Meanwhile, the DL group revealed a substantially inhibited development of severe root resorption lacunae compared to the DN group. Similar degree of slight surface resorption was shown in all groups, corresponding to the micro-CT analysis results.

## Discussion

This study focused on the effects of LIPUS treatment on replacement root resorption of avulsed teeth after replantation with different storage conditions and periods in dog models. 3D quantitative measurements of replacement root resorption and surface root resorption were carried out by micro-CT images to assess the effects of LIPUS on the healing of cementum and periodontal ligaments after a given trauma. In most previous studies, the amounts of root resorption were histologically measured^31^. Histological measurements help estimate the proportion of the root resorption area, but one should keep in mind that root resorption is not evenly distributed on the root surface and may preferentially affect corner surfaces^32,33^. If a single axial section is used as a reference of resorption, one section direction might show only little root resorption, whereas another section may demonstrate much more resorption.

Recently, 3D images of the microarchitecture of bone specimens from different species and body parts have been obtained by using micro-CT. This produces high resolution images by micro-CT and digitally reconstructs the tooth and surrounding alveolar bone, allowing for the quantitative assessment of root surface defects or bony attachments^34,35^. Nevins et al. showed that micro-CT can be used for the 3D quantitative assessment of alveolar bone^36^. De Paula Reis et al. showed, when compared to histologic analyses, that micro-CT in in vivo studies can be a promising method for the identification of the different stages of tooth resorption and repair, in addition to the evaluation of the total extension of the periodontium^37^. In this study, replacement root resorption, fusion between the alveolar bone and the root surface, and surface root resorption, craters or holes in the root surface, were measured in each coronal and cross-sectional image. The root resorption areas were reconstruced three-dimensionally and the percentages of resorption area to whole root surface were calculated. To the best of our knowledge, this is the first published report utilizing micro-CT to calculate the total surface area of a root to identify specific pathological parameters.

Dry storage for 60 min was used as a factor to increase damage to the periodontal ligament covering the root surface, according to the International Association of Dental Traumatology (IADT) guidelines for the management of avulsed permanent teeth where 60 min of extra-oral drying time is suggested as a criterion in making treatment plans and prognosis^38^. The length of dry extra-alveolar storage period is regarded as one of the strongest factors for PDL healing after tooth avulsion, affecting the survival of the PDL cells along the root surface. The guidelines indicate that after an extra-alveolar dry time of 30 min, most PDL cells are non-viable^39,40^. Andreasen et al. reported an increasing rate of ankylosis after delayed replantation over 5 min^41^. Maslamani et al. said that when the extra-oral dry time exceeds 60 min, it is considered that most PDL cells do not survive, and surface root resorption or dentoalveolar ankylosis of the tooth can progress further^42^. To exclude any potential factors that may influence or aggravate root resorption other than storage conditions, preoperative root canal treatment was performed and calcium hydroxide paste was added to minimize the progression of inflammatory root resorption due to infection of necrotic pulp^43^. In addition, decoronized roots were covered with gingiva as excessive initial force might cause severe root and bone resorption^44,45^. The lack of a negative control was based on the fact that an ideal storage medium would preserve the teeth in an environment that is similar to that of an immediate replantation (i.e. the positive control) and based on the extensive literature showing higher levels of replacement resorption for teeth that have been kept dry^8,46^. HBSS has been shown to be the best storage medium during the extra-oral phase^47^.

This study’s results showed that the surface resorption of the DN group was significantly higher than that of the HN and HL group (p < 0.05 and p < 0.01, respectively), possibly because of the low survival rates of PDL cells on the root surfaces, similar to those of previous studies^41,48^. In contrast, the DL group did not show any significant differences, whether with DN or with HN or HL. These results imply that LIPUS may positively affect regeneration of the periodontal tissue after intense avulsion conditions. However, since preventive root canal treatment measures were taken, the overall degree of surface resorption was minimal because of its self-limiting characteristics demonstrating repair with new cementum^49,50^. Most resorption lacunae are superficial and confined to the cementum, which is consistent with previous studies.

On the other hand, replacement resorption yielded more definite results. A significant reduction in replacement root resorption was found in the DL group compared to the DN group (p < 0.01). The HN and HL yielded similar outcomes. These results also implied that LIPUS may improve the recovery of cementum and periodontal tissue while necrotic PDL has undergone inflammatory change. These results are consistent with those of previous studies which showed that LIPUS accelerates the healing of resorption areas with the reparative cementum of orthodontic patients^51^ and stimulates the cementum regeneration of periodontal defects in canine models^35^. Furthermore, several in vitro studies demonstrate that LIPUS has an anabolic effect on human periodontal ligament cells by promoting mature cementoblasts^52^ and accelerating the differentiation of immature cementoblasts^53^. It has also been shown that LIPUS has the potential to accelerate endogenous periodontal mesenchymal stem cell recruitment for periodontal tissue regeneration on a molecular level^22^. It has been shown that ultrasound, if applied correctly, can stimulate tissue repair and wound healing^54,55^. Ultrasound during the inflammatory phase of tissue repair can lead to an acceleration of this phase, which eventually leads to an anti-inflammatory effect by LIPUS exposure^56^. Rego et al. suggested that LIPUS may contribute to reducing the inflammatory reaction by impairing the TNA-α signaling pathway^18,57^. They also demonstrated the inhibitory effect of a 21-day LIPUS application on root resorption using an experimental model of tooth replantation involving luxation and the immediate replacement of maxillary first molars in rats^18^.

Within the limitations of this animal study, both the 3D quantitative measurements of micro-CT and the histopathological examination demonstrated that LIPUS stimulation helped prevent the replacement resorption of avulsed teeth after delayed replantation. These results highlight the therapeutic implications of ultrasound stimulation in periodontal regeneration, suggesting the potential of LIPUS to inhibit dentoalveolar ankylosis. Additional studies are warranted to evaluate and capitalize on its full potential in the orofacial area. This animal study demonstrates one aspect of the application between therapeutic ultrasound and dental research. Considering that the ultrasound is actively used in medical fields, it could certainly contribute to the advancement of dental research.
